# Potential impact of climate change on the distribution of *Capricornis milneedwardsii*, a vulnerable mammal in China

**DOI:** 10.1002/ece3.11582

**Published:** 2024-06-25

**Authors:** Jiale Zhao, Weiwei Shao, Yalei Li, Haozhan Chen, Zhihua Lin, Li Wei

**Affiliations:** ^1^ College of Ecology Lishui University Lishui China; ^2^ College of Animal Science and Technology Zhejiang Agriculture and Forestry University Lin'an China; ^3^ College of Veterinary Medicine Zhejiang Agriculture and Forestry University Lin'an China

**Keywords:** *Capricornis milneedwardsii*, climate change, environmental variable, maximum entropy, potential distribution

## Abstract

Climate change significantly impacted on the survival, development, distribution, and abundance of living organisms. The Chinese serow *Capricornis milneedwardsii*, known as the “four unlike,” is a Class II nationally protected species in China. In this study, we predicted the geographical suitability of *C. milneedwardsii* under current and future climatic conditions using MaxEnt. The model simulations resulted in area under the receiver operating characteristic curve (AUC) values above 0.9 for both current and future climate scenarios, indicating the excellent performance, high accuracy, and credibility of the MaxEnt model. The results also showed that annual precipitation (Bio12), slope, elevation, and mean temperature of wettest quarter (Bio8) were the key environmental variables affecting the distribution of *C. milneedwardsii*, with contributions of 31.2%, 26.4%, 11%, and 10.3%, respectively. The moderately and highly suitable habitats were mainly located in the moist area of China, with a total area of 34.56 × 10^4^ and 16.61 × 10^4^ km^2^, respectively. Under future climate change scenarios, the areas of suitability of *C. milneedwardsii* showed an increasing trend. The geometric center of the total suitable habitats of *C. milneedwardsii* would show the trend of northwest expansion and southeast contraction. These findings could provide a theoretical reference for the protection of *C. milneedwardsii* in the future.

## Abstract 2

This study used the MaxEnt model to predict the current and future potential geographical distribution of *Capricornis milneedwardsii*, a vulnerable species endemic to China, under different climate change scenarios. Results suggest that the distribution of *C. milneedwardsii* is likely to expand in the future, with highly suitable areas increasing under both RCP 4.5 and RCP 8.5 scenarios. These findings could provide a theoretical reference for the protection of *C. milneedwardsii* in the future.

## 1 INTRODUCTION

Global climate change can lead to alterations in species distributions, increased extinction risk, and biodiversity loss (Purohit & Rawat, 2022; Raman et al., 2020; Zhao et al., 2024). To adapt to the impacts of future climate change, flora and fauna may substantially modify their geographical distributions (Purohit & Rawat, 2022; Pérez‐Suárez et al., 2024; Hou et al., 2023). Indeed, previous studies have demonstrated poleward and high‐elevation distribution shifts for some species as climate change accelerates, such as butterflies (Parmesan et al., 1999), birds (Thompson et al., 1993), fish (Yousefi et al., 2020), and Anura (Hou et al., 2023). Detailed and reliable spatial information regarding current species distributions is therefore essential to effectively model future ranges under climate change scenarios (Qin et al., 2017). To overcome issues with data incompleteness and predict future species range shifts, species distribution models (SDMs) are commonly utilized (Guan et al., 2022). SDMs are statistical tools that quantify the relationship between observed species occurrences and associated environmental conditions (Carvalho et al., 2011). Various modeling approaches exist for species distribution modeling. Some methods, such as the generalized additive model (GAM; Kosicki, 2018) and classification and regression tree (CART; Zhang et al., 2014), use presence–absence data. Others, like the genetic algorithm for rule set prediction (GARP; Padalia et al., 2014) and maximum entropy model (MaxEnt; Yang et al., 2023), leverage presence‐only records. Among these methods, MaxEnt has been widely adopted by researchers due to its ability to perform well with small sample sizes (Wisz et al., 2008), its use of presence‐only occurrence data and relevant environmental variables (Elith et al., 2011), and its direct output of habitat suitability maps (Purohit & Rawat, 2022). In recent years, MaxEnt has been successfully utilized to study habitat suitability for various animal and plant species, including *Graphium sarpedon* (Liao et al., 2024), *Panthera uncia* (Yang et al., 2023), *Tragopan caboti* (Zhao et al., 2023), *Cirsium lineare* (Fang et al., 2024), *Clerodendrum infortunatum* (Purohit & Rawat, 2022), and *Daphne mucronata* Royle (Abolmaali et al., 2018), with favorable outcomes. Predicting species distribution patterns and dynamic habitat suitability changes in advance is therefore crucial for developing protection and utilization strategies for at‐risk species (Chen et al., 2022).

**FIGURE 1:**
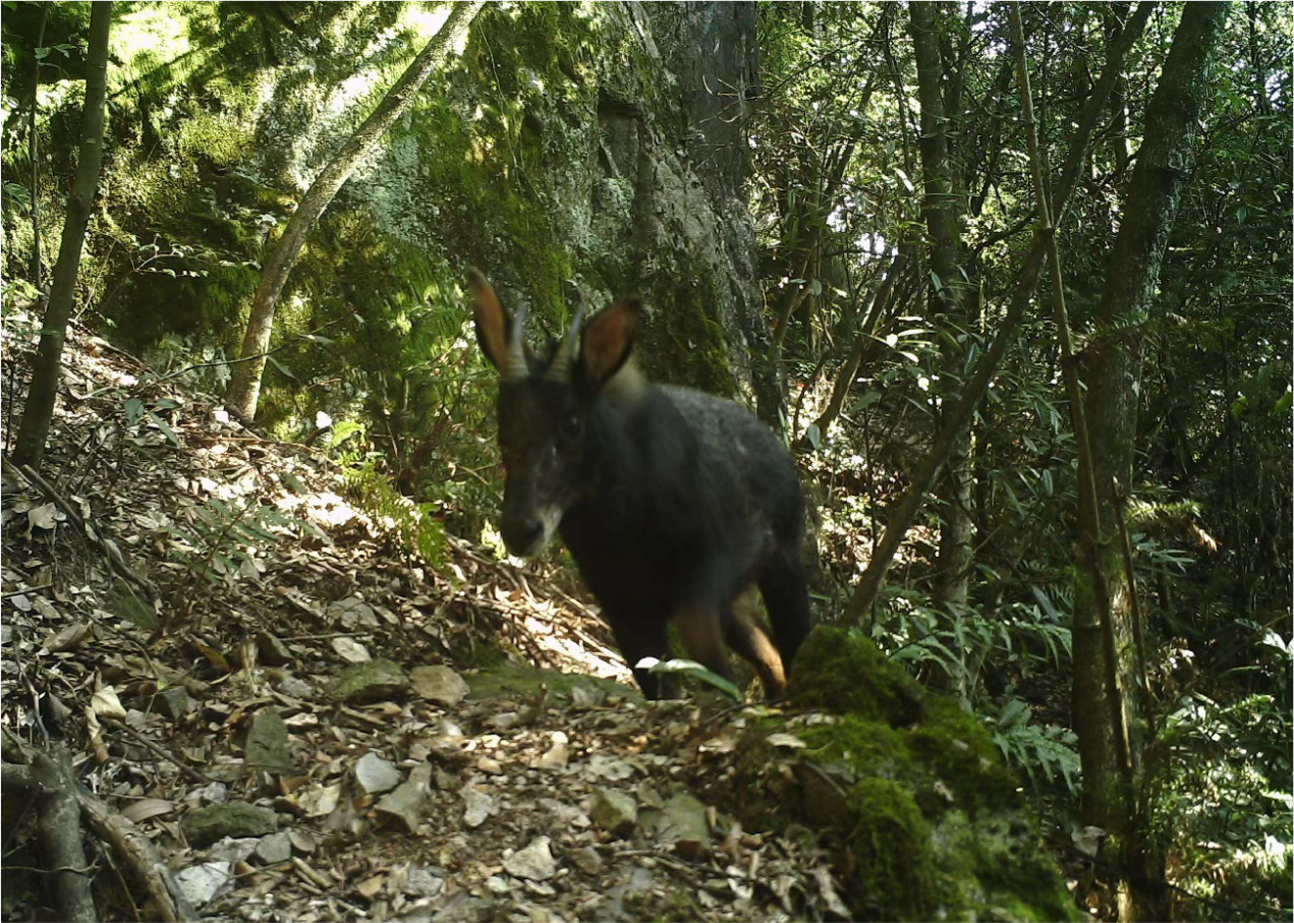
*Capricornis milneedwardsii*.

The Chinese serow *Capricornis milneedwardsii* is also known as “four unlike” because they look like deer but are not deer, have hooves like cattle but are not cows, have heads like sheep but are not sheep, and have tails like donkeys but are not donkeys (Figure 1). This species is native to China and Southeast Asia and has been listed as a Class II nationally protected species in China (Gong et al., 2016). Although it is widespread throughout its distributions, *C. milneedwardsii* is listed as a vulnerable species on the IUCN Red List (IUCN, 2011). This solitary and nocturnal species mainly inhabits coniferous and broad‐leaved mixed forests and natural karst scrublands (Liu, Guan et al., 2021). Its elusive lifestyle and rugged habitat make in‐depth study challenging (Liu, Lü et al., 2021). However, previous studies indicate that populations have become fragmented and are declining in many areas due to progressive habitat loss and overhunting (Francis, 2008; Thuc et al., 2014). Currently, habitat fragmentation and anthropogenic disturbances seriously threaten the survival of *C. milneedwardsii* (Liu et al., 2023). Urgent conservation interventions, such as strengthened habitat protections, are therefore needed.

In this study, we leveraged species location data compiled from literature searches and databases along with climate variables from WorldClim to map current and future potential distributions for *C. milneedwardsii* in China using MaxEnt modeling. We also aimed to determine key environmental factors restricting *C. milneedwardsii*'s range. Specifically, we evaluated changes in the geographical extent of suitable habitat between current and future climate scenarios.

## 2 MATERIALS AND METHODS

### 2.1 Collecting occurrence data of *C. milneedwardsii*

**FIGURE 2:**
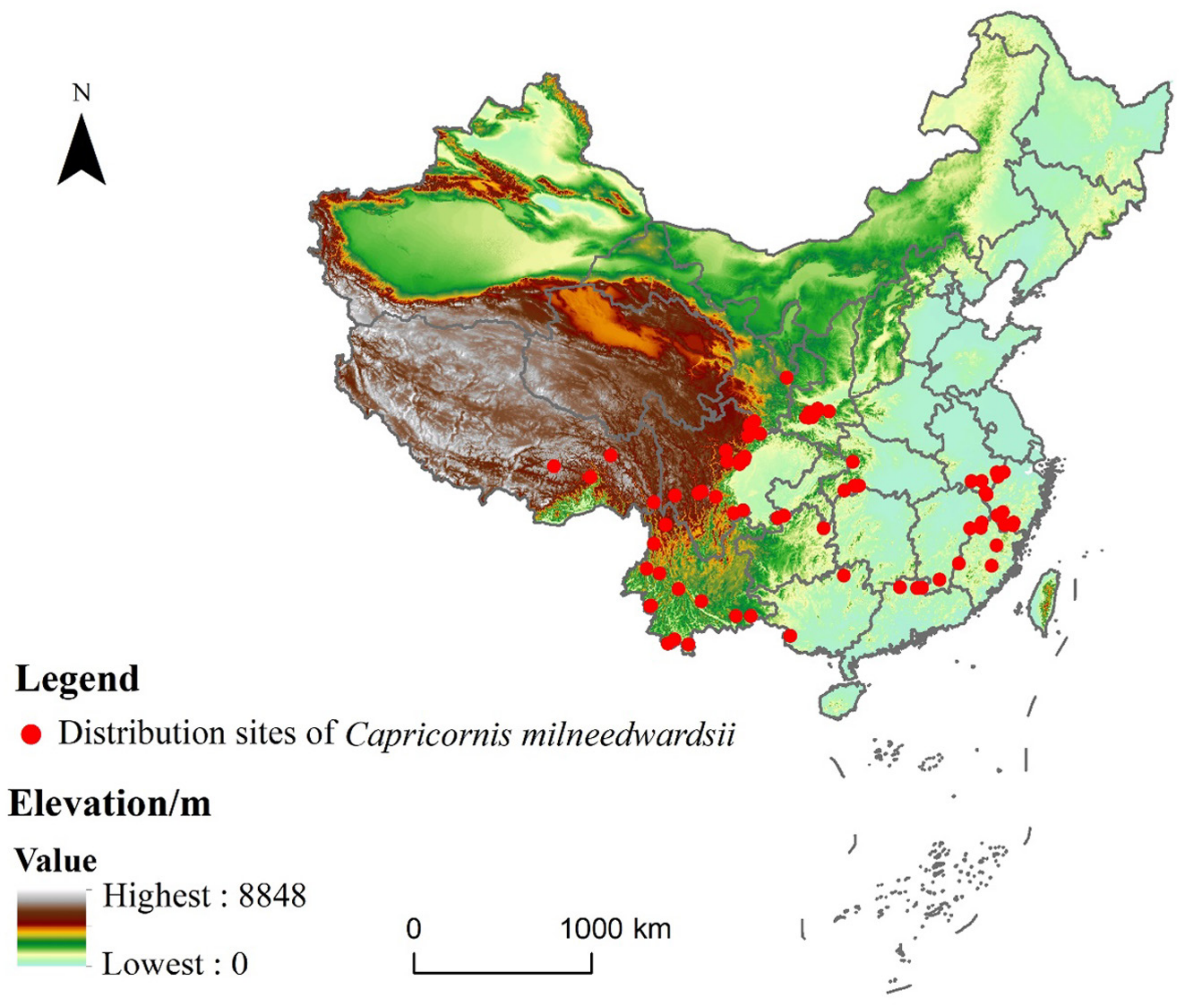
Geographical distribution of *Capricornis milneedwardsii* occurrence points.

Species distribution data were obtained from the Global Biodiversity Information Facility (GBIF) database, China National Knowledge Infrastructure (CNKI) database, Web of Science, and literature. After integrating the occurrence data from each source, the distribution information was depicted using R software (Chen, Guan et al., 2021). To ensure the accuracy, distribution point data were carefully screened. Data points that were repetitive, inaccurate, or significantly deviated from the species' known distribution range were eliminated following the previous research methods (Liu, Lü et al., 2021; Guan et al., 2022). To avoid overfitting the model, only one point was retained in each environmental factor grid with a spatial resolution of approximately 1 km × 1 km. ENMTools was used to randomly delete multiple coordinates that fell in the same grid based on the climate variables layer (Zhao et al., 2023). Finally, a total of 73 distribution points were obtained (Figure 2).

### 2.2 Environmental variables

To determine which environmental variables most influence the distribution of *C. milneedwardsii*, 19 bioclimatic variables and three topographic variables (elevation, slope and aspect) were selected. Following the methods described by Zhao et al. (2024), the climate data in this study were accessed through the Worldclim database (http://www.worldclim.org/) (accessed on 20 June, 2023) at 30 arc‐second (1 km × 1 km). The dataset included 19 bioclimatic variables that represent current temperature and precipitation conditions from 1970 to 2000 (Fick & Hijmans, 2017). To evaluate the impact of climate change on the species distribution, climate data for future predictions were also downloaded from the WorldClim database at 30 arc‐second (1 km × 1 km). The future climate data represented long‐term average climatic conditions in the 2050s (average for 2041–2060) and 2070s (average for 2061–2080) from the CCSM4 climatic system model released by CMIP6. The long‐term climate change projections are based on new scenarios known as representative concentration pathway (RCPs, IPCC, 2014). There are four RCP scenarios: RCP2.6, RCP 4.5/6.0 and RCP 8.5, which indicate low‐, medium‐, and high‐concentration greenhouse gas emission scenarios, respectively (Remya et al., 2015). Ultimately, RCP 4.5 and RCP 8.5 were selected to assess the distribution of suitable habitat of *C. milneedwardsii* in the future. The topographic data (elevation, slope, and aspect) were derived from digital elevation model (DEM) (http://www.gscloud.cn/) with a resolution of 1 km.

To reduce the risk of overfitting in the MaxEnt model due to environmental variables, pairwise Pearson's correlations among the variables were tested using ENMTools. Variables with |*r*| ≥ 0.8 were considered highly correlated, and the variables having the lower contribution value were retained. In the end, 13 environmental variables were selected for MaxEnt (Table 1).

**TABLE 1:** Environmental variables used in MaxEnt model and their percentage contribution.

Type	Code	Environmental variable	Unit	% contribution
Climate	Bio2	Mean diurnal range (Mean of monthly [max temp − min temp])	°C	6
	Bio3	Isothermality (Bio2/Bio7) (×100)		2.1
	Bio4	Temperature seasonality (standard deviation × 100)	C of V	0.6
	Bio6	Min temperature of coldest month	°C	2
	Bio7	Temperature annual range (Bio5–Bio6)	°C	5.1
	**Bio8**	**Mean temperature of wettest quarter**	**°C**	**10.3**
	**Bio12**	**Annual precipitation**	**mm**	**31.2**
	Bio14	Precipitation of driest month	mm	2.5
	Bio17	Precipitation of driest quarter	mm	0.9
	Bio19	Precipitation of coldest quarter	mm	0.8
Topography	**DEM**	**Elevation**	**m**	**11**
	**Slo**	**Slope**	**°**	**26.4**
	Asp	Aspect		1.1

### 2.3 Species distribution modeling

Based on the selected distribution data and environmental variables, the MaxEnt model was established and run 10 times. In each run, 75% of the data were randomly selected for model training, while the remaining 25% were used for model testing (Guan et al., 2022; Phillips, 2008; Zhao et al., 2023). Jackknife tests were performed to identify variables that reduce model reliability when omitted (Chen et al., 2022; Yang et al., 2023; Zhao et al., 2023). The area under the receiver operator curve (AUC) was used to evaluate model performance. An AUC value less than or equal to 0.7 indicates poor model performance, a value between 0.7 and 0.9 indicates moderate performance, and a value between 0.9 and 1.0 indicates excellent performance (Zhao et al., 2023, 2024).

For visualization and further analysis, the results of the Maxent models predicting the presence of *C. milneedwardsii* were imported into ArcGIS Pro (Reference ID: 602162530176). According to IPCC's explanation of the probability (*p*) of species' presence, potential habitats were divided into four categories: unsuitable habitat (*p* < .1), poorly suitable habitat (.1 ≤ *p* < .3), moderately suitable habitat (.3 ≤ *p* < .6), and highly suitable habitat (*p* ≥ .6). Following the methods of previous studies, the centroid of suitable areas under different climate change scenarios was calculated using the SDMtoolbox 2.4 in ArcGIS. Changes in centroid position under different scenarios were compared, and the migration distance of centroids was calculated (Chen et al., 2022; Guan et al., 2022; Zhao et al., 2023).

## 3 RESULTS

The MaxEnt model performed exceptionally well in predicting the distribution of *C. milneedwardsii* under current climate conditions, with an AUC value of 0.975 for the training data. The average training AUC values for the four future climate scenarios were also high, all exceeding 0.95, indicating the model's high accuracy and credibility (Table 2).

**TABLE 2:** Area under the receiver operating characteristic curve (AUC) under current climate and four future scenarios.

Years	Climatic scenario	Training AUC	Standard deviation
Current	–	0.975	0.004
2050	RCP4.5	0.970	0.007
	RCP8.5	0.974	0.007
2070	RCP4.5	0.970	0.006
	RCP8.5	0.973	0.007

**FIGURE 3:**
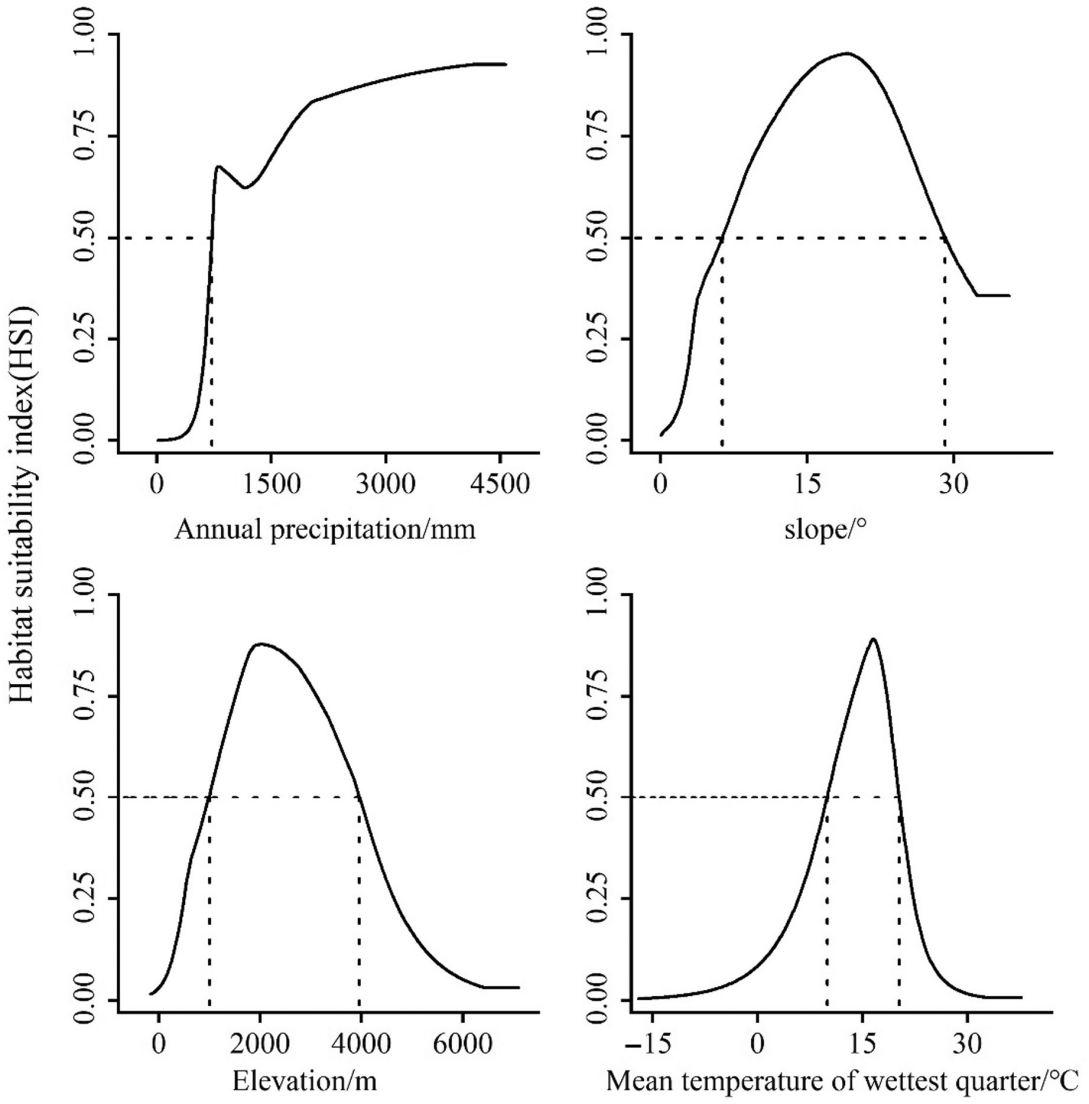
Response curve of dominant environmental factors.

Four environmental variables, namely annual precipitation (Bio12), slope, elevation, and mean temperature of the wettest quarter (Bio8), contributed 78.9% to the model's predictive power. Annual precipitation (Bio12) alone explained 31.2% of the total variance and was identified as the primary factor influencing the spatial distribution of *C. milneedwardsii* (Table 1). The species' response curves revealed that *C. milneedwardsii* prefers habitats with annual precipitation greater than 720.93 mm, slopes between 6.32 and 29.09 degrees, elevations ranging from 996.85 to 3953.99 m, and mean temperatures of the wettest quarter between 9.99°C and 20.23°C (Figure 3).

**FIGURE 4:**
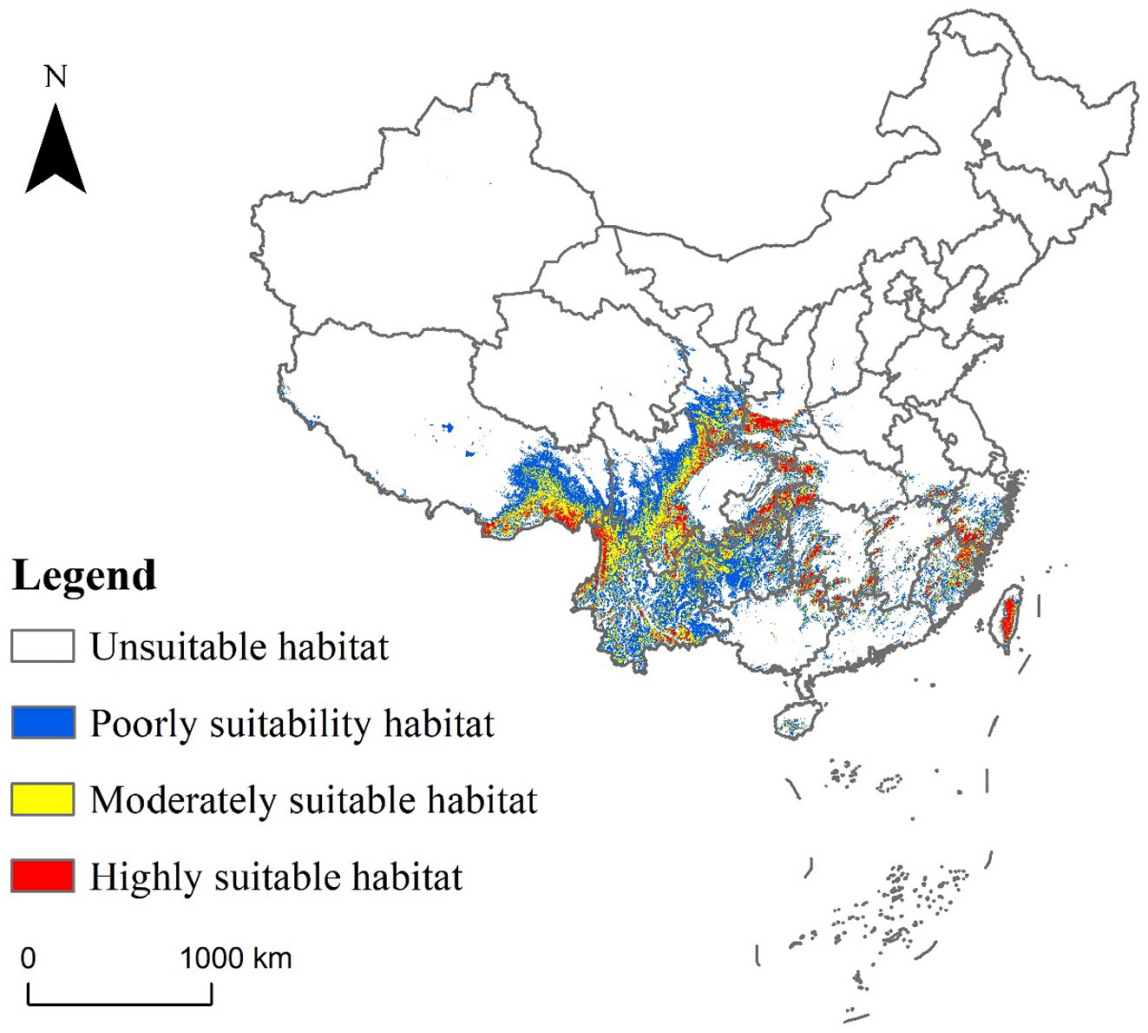
Distribution map of suitable habitat of *Capricornis milneedwardsii* under current climate.

The geographical distribution of *C. milneedwardsii* in China under current climate conditions, as predicted by MaxEnt, is shown in Figure 4. The suitable habitat for *C. milneedwardsii* is primarily located south of the Yellow River in mainland China and in the central region of Taiwan Province. The total suitable habitat area is approximately 123.21 × 10^4^ km^2^, representing about 12.83% of China's land area (Table 3). The moderately and highly suitable habitats are mainly distributed in southern Shaanxi, southeastern Gansu, central and western Sichuan, central and western Hubei, central and western Hunan, southeastern Tibet, northwestern Yunnan, northern Guizhou, southwestern Zhejiang, central Taiwan, and Fujian, with total areas of 34.56 × 10^4^ and 16.61 × 10^4^ km^2^, respectively (Figure 4). Within these suitable habitat areas, *C. milneedwardsii* is predominantly found in the moist regions of China.

**TABLE 3:** Potential habitat area (×10^4^ km^2^) of *Capricornis milneedwardsii* under current and future climate change scenarios.

Climate scenarios	Poorly suitable habitat	Moderately suitable habitat	Highly suitable habitat	Total area	Total change
Current	72.04	34.56	16.61	123.21	–
RCP4.5–2050	73.67	40.91	23.46	138.04	14.83
RCP8.5–2050	83.82	44.20	22.91	150.93	27.72
RCP4.5–2070	74.78	37.48	19.41	131.67	8.46
RCP8.5–2070	82.12	41.15	20.99	144.26	21.05

**FIGURE 5:**
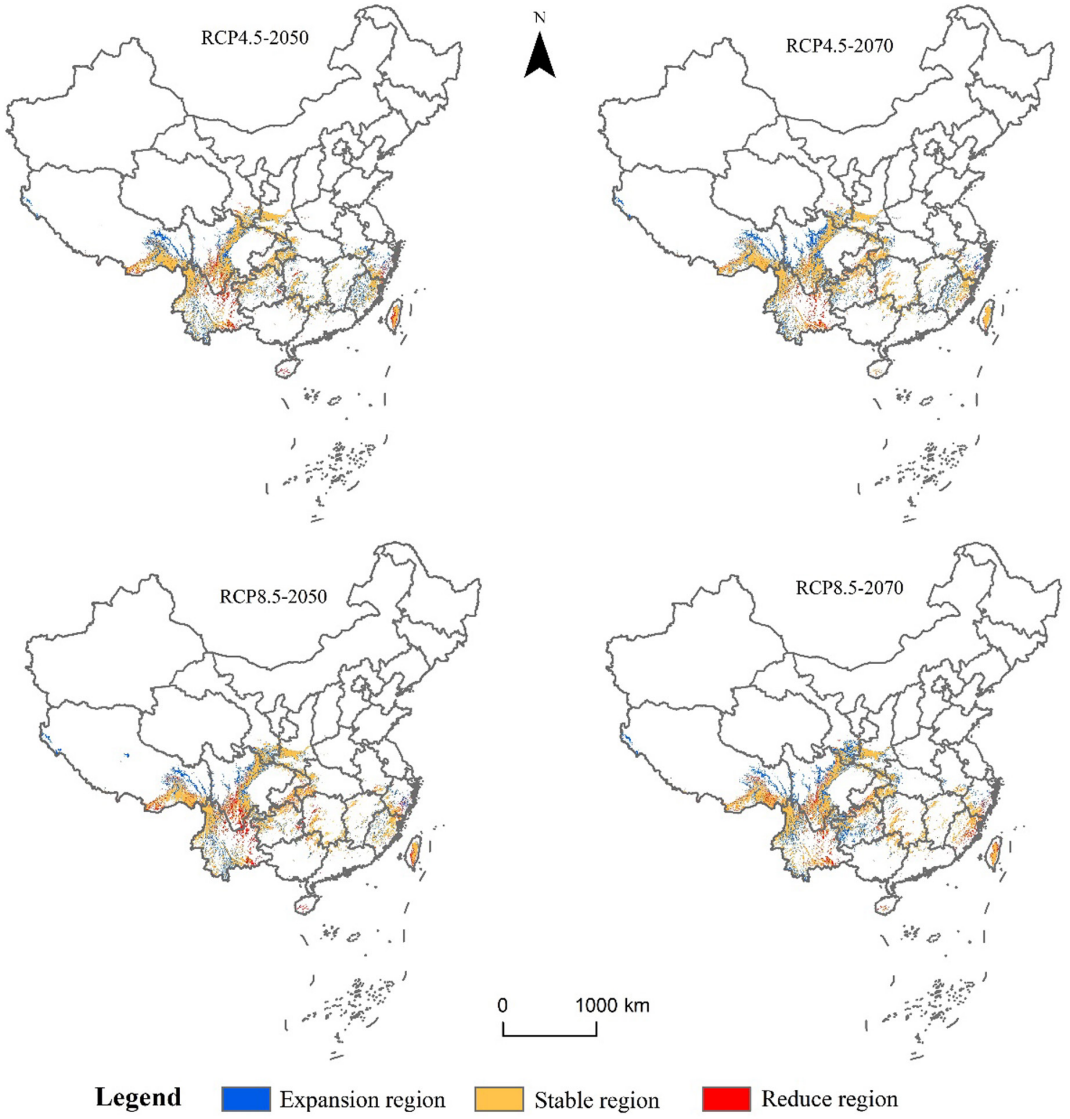
Changes in the distribution pattern of suitable habitat in highly and moderately of *Capricornis milneedwardsii* under different climatic conditions.

Under future RCP4.5 and RCP8.5 scenarios, the suitable habitat for *C. milneedwardsii* is projected to change (Figure 5). The areas of highly, moderately, and poorly suitable habitats are expected to increase significantly (Table 3). By the 2050s, the highly suitable habitat area is projected to increase to 23.46 × 10^4^ km^2^ under RCP4.5 and 22.91 × 10^4^ km^2^ under RCP8.5. The moderately suitable habitat area is expected to increase to 40.91 × 10^4^ km^2^ under RCP4.5 and 44.20 × 10^4^ km^2^ under RCP8.5. The poorly suitable habitat area is projected to increase to 73.67 × 10^4^ km^2^ under RCP4.5 and 83.82 × 10^4^ km^2^ under RCP8.5 (Table 3). The expansion of highly and moderately suitable habitats is mainly concentrated in southeastern Tibet, central and western Yunnan, central Sichuan, southwestern Zhejiang, central and northern Fujian, with a small increase in northwestern Tibet. In contrast, reductions in highly and moderately suitable habitats are primarily observed in eastern Yunnan and south‐central Sichuan (Figure 5). By the 2070s, the highly suitable habitat area is projected to increase to 19.41 × 10^4^ km^2^ under RCP4.5 and 20.99 × 10^4^ km^2^ under RCP8.5. The moderately suitable habitat area is expected to increase to 37.48 × 10^4^ km^2^ under RCP4.5 and 41.15 × 10^4^ km^2^ under RCP8.5. The poorly suitable habitat area is projected to increase to 74.78 × 10^4^ km^2^ under RCP4.5 and 82.12 × 10^4^ km^2^ under RCP8.5 (Table 3).

**FIGURE 6:**
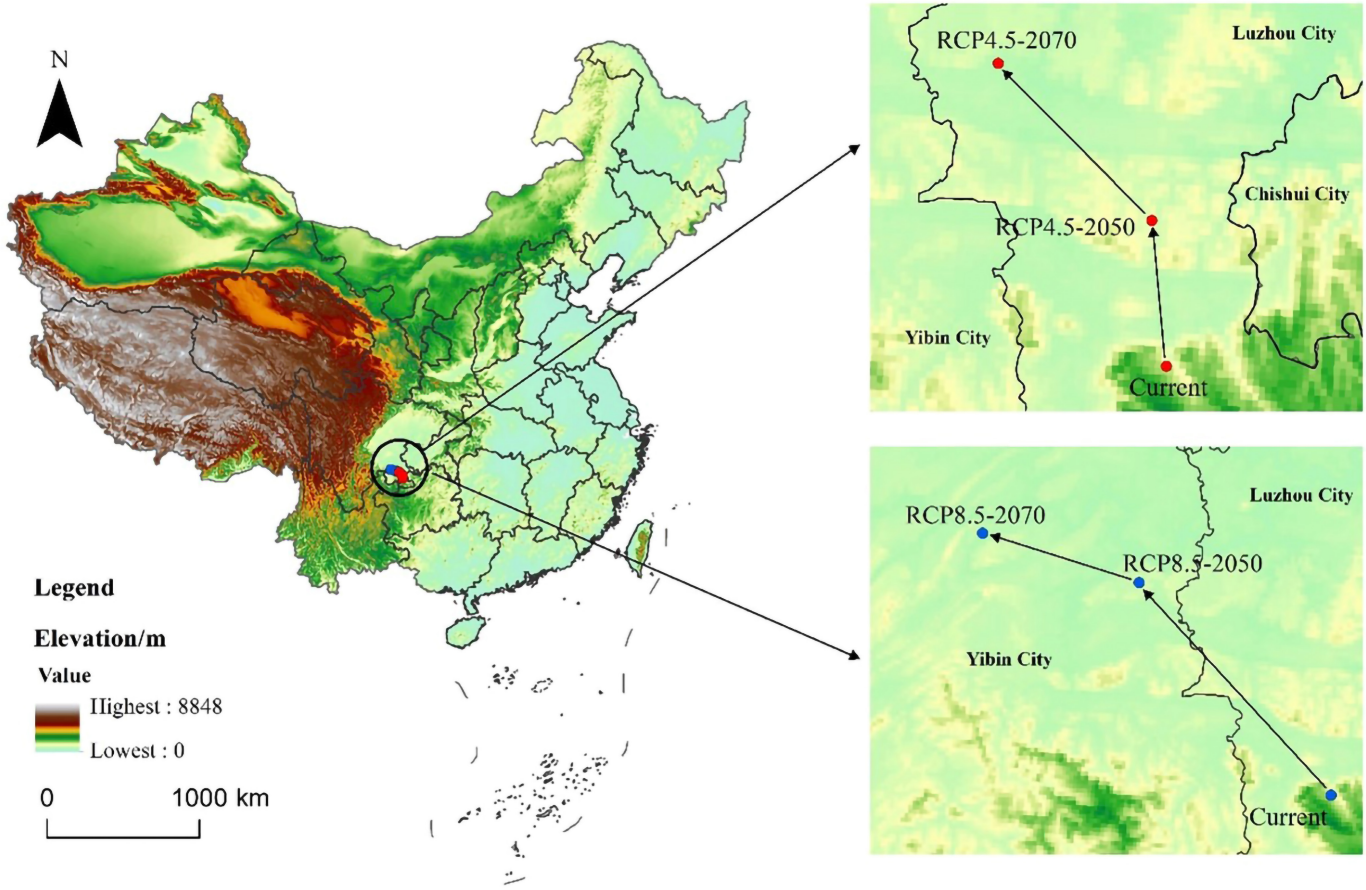
Suitable location of geometric center for *Capricornis milneedwardsii* under different climate scenarios.

Under current climate conditions, the geometric center of the potential suitable habitat for *C. milneedwardsii* is located in the southeast of Sichuan Province, China (Figure 6). By the 2050s, under RCP4.5, the geometric center is projected to shift 19.31 km northward from Xuyong (105°31′55.94″ E, 28°16′3.90″ N) to the north of the county (105°30′17.06″ E, 28°26′26.83″ N). By the 2070s, the geometric center is expected to further move 28.94 km northwestward to Naxi (105°17′7.67″ E, 28°36′57.44″ N) (Figure 6). Under RCP8.5, by the 2050s, the geometric center is projected to shift 62.85 km northwestward from Xuyong to Jiang'an (105°4′33.85″ E, 28°40′3.11″ N). By the 2070s, the geometric center is expected to move an additional 36.18 km northwestward to Cuiping (104°43′0.75″ E, 28°44′55.08″ N) (Figure 6).

## 4 DISCUSSION

### 4.1 Performance of the MaxEnt model

The MaxEnt model has proven to be a robust tool for studying the spatial distribution of species (Elith et al., 2006). In this study, we employed the MaxEnt model to predict the current and future potential distributions of *C. milneedwardsii* using bioclimatic and topographic variables. Our model achieved a high AUC value of 0.975, surpassing those reported for small‐scale studies of this species by Feng et al. (2022); AUC = 0.878, Liu et al. (2023); AUC = 0.944, and Meng et al. (2023); AUC = 0.852. The variable AUC accuracy across scales highlights the limitations of small‐scale niche modeling and validates our large‐scale approach. Comparable evaluations for other species also demonstrate a range in MaxEnt prediction success, with reported AUC values of 0.915 for *Ailuropoda melanoleuca* in the Minshan Mountains (Chen, Zhu et al., 2021), 0.970 for *Chrysolophus pictus* in central and western China (Ye et al., 2021), and 0.923 for *Naemorhedus griseus* in the Qinling Mountains (Liu et al., 2022).

### 4.2 Key environmental factors

Our results indicate that the suitable habitat of *C. milneedwardsii* is mainly distributed south of the Yellow River in mainland China and in the central area of Taiwan Province. These areas are located in the tropical and subtropical regions of Southeast Asia with a humid climate (Sheng & Lu, 1985), which provides abundant food resources and habitat conditions that affect the growth and development of *C. milneedwardsii* (Liu et al., 2023). We found a strong coupling between habitat suitability and annual precipitation (Bio12), slope, elevation, and mean temperature of the wettest quarter (Bio8). The response curves showed that the presence probability of *C. milneedwardsii* increased when annual precipitation exceeded 720.93 mm and the mean temperature of the wettest quarter ranged from 9.99 to 20.23°C, indicating that the species' presence is strongly depends on rainfall and temperature. Additionally, *C. milneedwardsii* tends to prefer medium–high‐elevation gradients, with a higher probability of occurrence at elevations ranging from 996.85 to 3953.99 m, which is consistent with previous studies by Feng et al. (2022) and Liu et al. (2023). The combination of different latitudes and elevations may form similar local climatic and hydrothermal conditions, thus creating suitable habitat conditions for *C. milneedwardsii*. Furthermore, habitat suitability showed a significant correlation with slope, which may be attributed to the species' habit of basking on bare rock and using steep mountains for escape, as well as its extensive food habits (Liu et al., 2023; Wu & Hu, 2001).

Previous studies focusing specifically on Gongga Mountain (Liu et al., 2023), Fanjingshan National Nature Reserve (Meng et al., 2023), and the Minshan, Qionglai, and other Sichuan mountain systems (Feng et al., 2022) identified differing environmental factors as key drivers of *C. milneedwardsii* distribution. For example, Liu et al. (2023) found that precipitation seasonality (Bio15), vegetation, isothermality (Bio3), elevation, and distance to rivers most influenced local *C. milneedwardsii* distributions. These discrepancies in results may be explained by several factors. Firstly, our study only considered bioclimatic and topographic data, while Feng et al. (2022) and Liu et al. (2023) integrated additional variables like vegetation indices and proximity to water and human activity into their models. Secondly, our study used large‐scale occurrence data, whereas Feng et al. (2022) and Liu et al. (2023) based their analyses on localized presence records. To elucidate the most influential drivers of *C. milneedwardsii* distribution, future modeling efforts should incorporate more comprehensive environmental predictors and evaluate a multi‐scale approach.

### 4.3 Habitat requirements and potential impacts of climate change

Forest type comprehensively reflects the characteristics of food composition, temperature, light, topography, and landform that animals require, and it meets the needs of animals for habitat selection to the greatest extent (Wu & Hu, 2001). Previous studies have shown that the main habitats of *C. milneedwardsii* are broad‐leaved forests and coniferous broad‐leaved mixed forests (Liu et al., 2023; Sun et al., 2007). The breeding season of *C. milneedwardsii* occurs in late October and November, with a single kid born after a 7‐month gestation period. The kid may stay with its mother for almost a year (Thuc et al., 2014), and *C. milneedwardsii* reaches reproductive maturity after 2–3 years, around late May or early June (Francis, 2008). Moreover, this species tends to inhabit rugged, steep hills, and rocky terrain (Francis, 2008). Therefore, suitable habitats that provide food abundance, concealment, and appropriate temperatures likely play important roles in the survival and dispersal of *C. milneedwardsii*.

Climate is a key factor affecting the distribution of species (Castex et al., 2018). In the future, global warming may cause the subtropical monsoon climate boundary to move northward, potentially providing new suitable habitats for *C. milneedwardsii* and inducing upward and northward distributional shifts from lower to higher elevations and latitudes. Indeed, our modeling results showed that the highly suitable area for *C. milneedwardsii* will increase under both RCP 4.5 and RCP 8.5 scenarios compared with the current potential distribution. These results align with previous studies demonstrating how climate change could shift species distributions poleward and to higher elevations, as observed for *Aspidoscelis costata costata* (Güizado‐Rodríguez et al., 2012), *Inachis io* (Ryrholm, 2003), and smallmouth bass (Chu et al., 2005).

### 4.4 Conservation implications and recommendations

Our study underscores the significance of China's moist areas as the primary habitats for *C. milneedwardsii* and highlights the need to prioritize these regions, known for their high levels of wildlife diversity and activity (Liao et al., 2024), in conservation efforts. The MaxEnt model developed here identifies temperature, rainfall, elevation, and slope as the key environmental factors influencing the species' habitat suitability, providing valuable insights for targeted conservation strategies.

We recommend the following actions: (1) Conservation authorities should focus on monitoring and managing these specific environmental variables to ensure the persistence of suitable habitats for *C. milneedwardsii*. Long‐term monitoring of vegetation status and climate factors in the species' habitats is crucial for understanding the impacts of climate change and other anthropogenic pressures, and for developing adaptive management strategies. (2) A combination of GPS collars, infrared camera monitoring techniques, metagenomics, and other macrocosm and microcosm approaches should be employed to comprehensively study the conservation ecology of *C. milneedwardsii*. (3) Different types of protected areas, such as national parks, nature reserves, and natural parks, should be established and strengthened as important forms of in situ conservation for *C. milneedwardsii*. (4) Based on a comprehensive database of species diversity and considering environmental factors such as climate, vegetation, terrain, and human activities, a variety of niche models should be used to construct spatially explicit maps of suitable habitat for the species. (5) Laws and regulations on wildlife protection should be improved and strictly enforced, with efforts made to raise public awareness and resolutely curb illegal hunting and trade in wildlife products.

Our research contributes to the growing knowledge on the ecological requirements and potential vulnerabilities of *C. milneedwardsii*, providing a foundation for evidence‐based conservation planning. We urge policymakers to utilize these findings to formulate and implement effective, science‐driven strategies for the protection of *C. milneedwardsii* and its critical habitats, ensuring the long‐term viability of this iconic species in the face of ongoing environmental challenges.

## 5 CONCLUSION

This study used the MaxEnt model to predict the current and future potential geographical distribution of *C. milneedwardsii*, a vulnerable species native to China, under different climate change scenarios. Results suggest that the distribution of *C. milneedwardsii* is likely to expand in the future, with highly suitable areas increasing under both RCP 4.5 and RCP 8.5 scenarios. Annual precipitation, slope, elevation, and mean temperature of the wettest quarter were identified as the most important environmental variables, contributing 78.9% to the predictions.

The habitat suitability maps generated in this study can inform conservation planning and management of *C. milneedwardsii* by prioritizing areas for monitoring, protection, and habitat restoration efforts. However, the model's limitations include the lack of consideration for other potentially important environmental variables such as interspecific competition, distance to water sources, vegetation types, and anthropogenic factors. Future research should integrate these additional variables to achieve more comprehensive predictions. Despite these limitations, our study provides valuable insights for the conservation and management of *C. milneedwardsii*. We recommend that conservation authorities and policymakers use this knowledge to develop evidence‐based strategies for the protection of this vulnerable species and its critical habitats. As climate change continues to threaten biodiversity, studies like ours will become increasingly important for guiding proactive, science‐based conservation strategies.

## AUTHOR CONTRIBUTIONS

**Li Wei:** Conceptualization (equal); supervision (equal). **Jiale Zhao:** Investigation (equal); methodology (equal); software (equal). **Weiwei Shao:** Investigation (equal); writing – original draft (equal). **Yalei Li:** Investigation (equal); writing – review and editing (equal). **Haozhan Chen:** Investigation (equal); visualization (equal). **Zhihua Lin:** Investigation (equal); resources (equal); software (equal).

## FUNDING INFORMATION

This study was supported by the Key Research Projects of Lishui City (2021ZDYF05) and project commissioned by Jinyun County Forestry Administration (20230801).

## CONFLICT OF INTEREST STATEMENT

The authors declare that they have no competing interests.

## Data Availability

The datasets generated and/or analyzed during the current study are available in a public repository at: GBIF. org (May 20, 2023) GBIF Occurrence Download https://www.gbif.org/. The climate data in this study were accessed through the Worldclim database (http://www.worldclim.org/) (accessed on June 20, 2023). The future climate data represented long‐term average climatic conditions in 2050s (average for 2041–2060) and 2070s (average for 2061–2080) from the CCSM4 climatic system model that released by CMIP6 (http://www.worldclim.org/). The topographic data (elevation, slope, and aspect) were derived from digital elevation model (DEM) (http://www.gscloud.cn/).
